# Folded bandage contact lens retention in a patient with bilateral dry eye symptoms: a case report

**DOI:** 10.1186/s12886-017-0505-4

**Published:** 2017-07-04

**Authors:** Derek K.-H. Ho, John P. Mathews

**Affiliations:** Stanley Eye Unit, Abergele Hospital, Llanfair Road, Abergele, Conwy, North Wales, LL22 8DP UK

**Keywords:** Case report, Bandage contact lens, Upper fornix, Lid eversion

## Abstract

**Background:**

Bandage contact lenses are commonly used by ophthalmic practitioners to protect the patient’s cornea. We report a case of folded bandage contact lens retained for six and a half years in the upper subtarsal space. To our knowledge, no other cases of retained bandage contact lens have previously been reported in the literature.

**Case presentation:**

A patient was applied a pair of bandage contact lenses due to persistent ocular pain secondary to dry eye symptoms. At her subsequent visit, bandage contact lens was removed from her left eye, but none was found in the right eye. Documentation from further visit stated that the bandage contact lenses were no longer in situ. 6.5 years since the lens insertion, lid eversion revealed a ‘foreign body’ retained beneath her right upper eyelid, which was noted to be a folded, discoloured bandage contact lens.

**Conclusions:**

The ‘upper fornix trap’, where the contact lens may be retained by the upper tarsal edge, presents an anatomical hazard for contact lens users. Moreover, soft contact lenses may be more likely to retain asymptomatically and to fold onto itself compared to hard lenses. Our case report highlights the importance of performing a thorough eye examination, which includes double eversion of the upper eyelids and sweeping of the fornices with cotton buds, and maintaining clinical suspicion of contact lens retention.

## Background

Bandage contact lenses (BCLs) are commonly used in ophthalmic departments to protect the cornea. BCLs can be available at larger diameters than modern commonly-used corrective soft contact lenses [1, 2]. Larger diameter reduces the contact lens’ movement, which is important in protecting a diseased cornea [3].

The conjunctiva lining the posterior surface of the eyelid divided into palpebral conjunctiva (on the under-surface of the eyelids), bulbar conjunctiva (over the eyeball up to the limbus) and conjunctival fornix (the cul de sac where the conjunctiva turns on itself between the palpebral and bulbar zones). Occasionally foreign bodies, including contact lenses, may be hidden at the posterior aspect of the palpebral conjunctiva near the fornix, only to be discovered by the examiner when the upper eyelid is everted. The ‘upper fornix trap’ was first described by Bock in 1971 [4], who postulated that the contact lens can become ‘trapped’ within the upper conjunctival fornix, with the lens’ lower border wedged into the upper tarsal edge. There have been published cases on retained contact lenses presenting as mass, cyst or chalazion [5–9]. We report a case of folded bandage contact lens retained for six and a half years in the upper subtarsal space of an elderly patient, who had a long history of bilateral dry eye symptoms. To our knowledge, no other cases of retained bandage contact lens have previously been described in the literature.

## Case presentation

An eighty-two-year-old female patient, with a background of longstanding dry eye symptoms having undergone bilateral cataract surgeries a few months prior, presented to the hospital eye casualty clinic complaining of foreign body sensation. The examining doctor performed a detailed anterior segment examination and diagnosed her with bilateral meibomian gland dysfunction. She was given lid hygiene instructions together with antibiotic and lubricating eyedrops for a month.

When patient presented to the clinic a year later, in view of the persistent bilateral ocular pain a pair of Precision UV© (base curve 8.7 mm; diameter 14.4 mm; Contents: Vasurfilcon A 26% and water 74%) BCLs were inserted. Four weeks later at the follow-up visit, another ophthalmologist removed the BCL from her left eye, while none was found in the right eye. No further BCLs were placed. She returned six months later still complaining of burning sensation in both eyes. There were no foreign bodies noted, although no upper eyelid eversion was documented. She was treated for dry eyes. Six more consultations took place over the next 4 years with different ophthalmologists within the same eye unit, during which the examinations uncovered bilateral superficial punctate keratitis but no eyelid lump or any other observable anterior segment abnormalities. Various treatment strategies including punctal plugs, several lubricant regimens such as Celluvisc© 0.5%, HYLO-Tear© and Lacri-lube© were prescribed for her bilateral dry eye symptoms but none were successful.

**Fig. 1:**
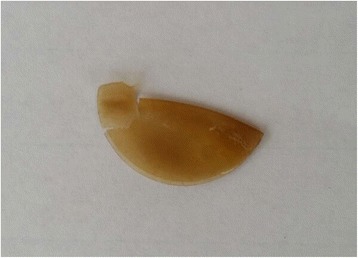
Folded, discoloured bandage contact lens hidden behind the patient’s upper eyelid

It was not until six and a half years after the BCL insertion, when a nurse practitioner from the eye casualty unit everted her right upper eyelid and discovered a ‘foreign body’, noted to be a folded, discoloured bandage contact lens [Fig. 1], which was removed without difficulty. Three months later, unfortunately, she still struggles with bilateral dry eyes, despite the daily use of lubricating eyedrops.

## Discussion and Conclusions

The ‘upper fornix trap’, first coined by Bock in 1971 [4], described a ‘trapped’ contact lens within the upper conjunctival fornix, where the lens’ lower border is wedged into the upper tarsal edge. At such position, there is a possibility of contact lens erosion from the upper fornix towards subconjunctival space, promoted by pressure necrosis of the surrounding tissue. Erosion of such foreign bodies into eyelid tissue could even clinically present as a cyst or chalazion [5–9].

A diagnosis of retained contact lens may be missed when the clinical picture is masqueraded by other distracting information, such as longstanding dry eye symptoms and chronic conjunctivitis [7]. In our case, the retained bandage contact lens remained undetected for six and a half years despite several clinical visits during the period. The lack of clinical suspicion may be due to the bilateral nature of her ocular complaint and the absence of unilateral symptoms such as foreign body sensation, discomfort or periocular swelling, which led the clinicians away from considering contact lens retention. Without an observable eyelid lump and without performing lid eversion, the clinicians only noticed superficial punctate keratitis on examination, which could have been explained by her dry eye condition.

It has previously been suggested that soft contact lenses may have a higher possibility of asymptomatic retention than hard lenses [5]. In our case, we hypothesize that retained soft contact lenses may also more readily fold onto itself spontaneously, with the help of blinking movement. The higher risks associated with soft contact lens, combined with patients who may not necessarily offer a history of misplaced contact lens, highlight the importance of performing a thorough eye examination, which includes double eversion of the upper eyelids and sweeping of the fornices with cotton buds, to reliably rule out the possibility of any retained contact lenses.

## Abbreviation

BCL: Bandage contact lens

## References

[1] Young M. Whats ahead in 2010: Bandage contact lenses http://www.eyeworld.org/article-bandage-contact-lenses,2. Accessed 2 Nov 2016.

[2] Young G, Potts M, Sulley A (2016). The effect of temperature on soft contact lens diameter. Eye Contact Lens.

[3] McDermott ML, Chandler JW (1989). Therapeutic uses of contact lenses. Surv Ophthalmol.

[4] Bock RH (1971). The upper fornix trap. Br J Ophthalmol.

[5] Agarwal PK, Ahmed TY, Diaper CJ (2013). Retained soft contact lens masquerading as a chalazion: a case report. Indian J Ophthalmol.

[6] Shams PN, Beckingsale AB, Sheldrick JH, Rose GE. An unusual eyelid lump: unsuspected embedded contact lens for up to 40 years. Two cases and literature review. Eye (Lond). 2011;25(10):1371–3. 10.1038/eye.2011.136 PMC3194303 21720414

[7] Zola E, van der Meulen IJ, Lapid-Gortzak R, van Vliet JM, Nieuwendaal CP (2008). A conjunctival mass in the deep superior fornix after a long retained hard contact lens in a patient with keloids. Cornea.

[8] Benger RS, Frueh BR (1986). An upper eyelid cyst from migration of a hard corneal contact lens. Ophthalmic Surg.

[9] Nicolitz E, Flanagan JC (1978). Orbital mass as a complication of contact lens wear. Arch Ophthalmol.

